# The DySLOH Study: Comparative Evaluation of the Results between the ProFlor and Lichtenstein Techniques for Open Inguinal Hernia Repair—A Randomized Controlled Trial

**DOI:** 10.3390/jcm13185530

**Published:** 2024-09-18

**Authors:** Giorgio Romano, Giuseppe Di Buono, Vito Rodolico, Giorgio Romano, Gabriele Barletta, Guido Zanghì, Pietro Giorgio Calò, Salvatore Buscemi, Antonino Agrusa

**Affiliations:** 1 Department of Precision Medicine in Medical, Surgical and Critical Care (Me.Pre.C.C.), University of Palermo, 90127 Palermo, Italy; giorgio.romano@unipa.it (G.R.); salvatore.buscemi02@unipa.it (S.B.); antonino.agrusa@unipa.it (A.A.); 2 Department PROMISE, Section Pathological Anatomy, University of Palermo, 90127 Palermo, Italy; vito.rodolico@unipa.it; 3 Postgraduate School of General Surgery, University of Palermo, 90127 Palermo, Italy; giorgioromano95@gmail.com (G.R.); gabrielebarletta@gmail.com (G.B.); 4 Department of General Surgery, University of Catania, 95124 Catania, Italy; gzanghi@unict.it; 5 Department of Surgical Sciences, University of Cagliari, 95124 Cagliari, Italy; pgcalo@unica.it

**Keywords:** hernia, inguinal hernia, hernia surgery, mesh, dynamic mesh

## Abstract

**Background:** The Lichtenstein open anterior approach with static flat meshes, the most popular inguinal hernia repair technique, has raised concerns regarding mesh fixation, defect patency, and poor quality biological response. To address these issues, the 3D dynamic ProFlor scaffold promoting a fixation-free hernia defect obliteration has been developed as an alternative. **Methods:** The results of open inguinal hernia repair with the ProFlor approach compared with those of the Lichtenstein repair were evaluated. **Results:** In a time frame of 24 months, two cohorts of patients were enrolled, 95 in the ProFlor group and 93 in the Lichtenstein group. ProFlor demonstrated superior outcomes compared to the Lichtenstein technique, with shorter procedure times, decreased intraoperative complications, and lower rates of postoperative complications. Additionally, ProFlor provided enhanced postoperative pain relief, a faster return to daily activities, and no long-term discomfort. No chronic pain was reported in the ProFlor group and 11.8% reported chronic pain in the Lichtenstein group. **Conclusions:** The results highlight the need to reevaluate the conventional Lichtenstein approach and align it with recent scientific progress. Further consideration of the evolving understanding of inguinal pathophysiology and groin protrusion genesis is crucial for advancing surgical techniques.

## 1. Introduction

Inguinal herniorrhaphy is a widely performed surgical procedure worldwide, with millions of surgeries being conducted each year, including over 800,000 procedures in the United States alone [1]. Over the centuries, numerous techniques have been proposed for the treatment of inguinal hernias. Lichtenstein made further advancements in the 1960s by developing the “tension-free hernia repair” technique [2,3]. This technique aimed to repair the hernia without placing tension on the surrounding tissues, reducing the risk of recurrence and improving patient outcomes. The Lichtenstein inguinal hernia repair technique has been further developed to include the use of static plugs inserted into the hernial defect to fill the gap [4]. This modification is known as the mesh and plug technique. While there is ongoing debate and variation in surgical management and the choice of prosthetics, the Lichtenstein technique continues to be widely used as the primary method of treatment for inguinal hernias. Indeed, despite advancements in surgical techniques and the use of prosthetic materials, complications such as bleeding, hematoma, infection, and recurrence are associated with prosthetic repair and affect postoperative outcomes of inguinal herniorrhaphy [5,6]. One area of concern is the interaction between the procedural steps of the repair technique and the prosthetic material used. The choice of technique, the type of mesh, and the method of fixation can all influence the occurrence of adverse events [7]. Complications specific to prosthetic inguinal herniorrhaphy can arise, which may include implant-related issues and poor biological response to the mesh [8]. One significant concern is the development of long-lasting postoperative pain, discomfort, or even chronic pain syndrome [9,10,11,12]. When evaluating the Lichtenstein technique and similar approaches for inguinal hernia repair from a physiological and pathogenetical perspective, it becomes apparent that certain aspects of these surgical treatments may not align with the natural physiology of the groin and the underlying degenerative process of the disease. The fixation of mesh onto the delicate and highly mobile myotendineal structures of the groin may not be in harmony with the physiological dynamics of the inguinal region and may impair the kinetic of the groin. Additionally, the foreign body reaction induced by biocompatible static meshes results in a granulomatous response [13,14]. While this reaction may not be detrimental in all cases, it deviates from the ideal goal of tissue regeneration and restoration that should be targeted to address the underlying degenerative nature of inguinal hernia [15]. Based on these considerations, a novel device has been developed for repairing inguinal hernias. Named ProFlor (produced under license by Insightra Medical Inc., Clarksville, TN, USA), it is constructed from polypropylene, similar to conventional prostheses, and features a 3D multilamellar structure with centrifugal expansion. This design enables the device to be deployed without the need for fixation, ensuring a secure and permanent obliteration of the hernial defect [16]. Due to its inherent dynamic compliance, the device responds to the natural movements of the groin, effectively adapting to the physiological squeezing and relaxing of the inguinal musculature. In addition, the dynamic responsivity of ProFlor has been demonstrated to attract a wide range of tissue growth factors, promoting the incorporation of viable tissue within its 3D structure like a regenerative scaffold [17,18]. Over the past few years, ProFlor has gained widespread adoption for inguinal hernia repair, demonstrating promising outcomes in thousands of patients operated worldwide [19]. However, to establish the true efficacy of this innovative technique, a comparative study is deemed necessary, focusing on the intra- and postoperative outcomes of the open anterior ProFlor approach in comparison to the conventional Lichtenstein technique, which remains the most commonly performed open hernia repair method.

## 2. Methods

**Figure 1:**
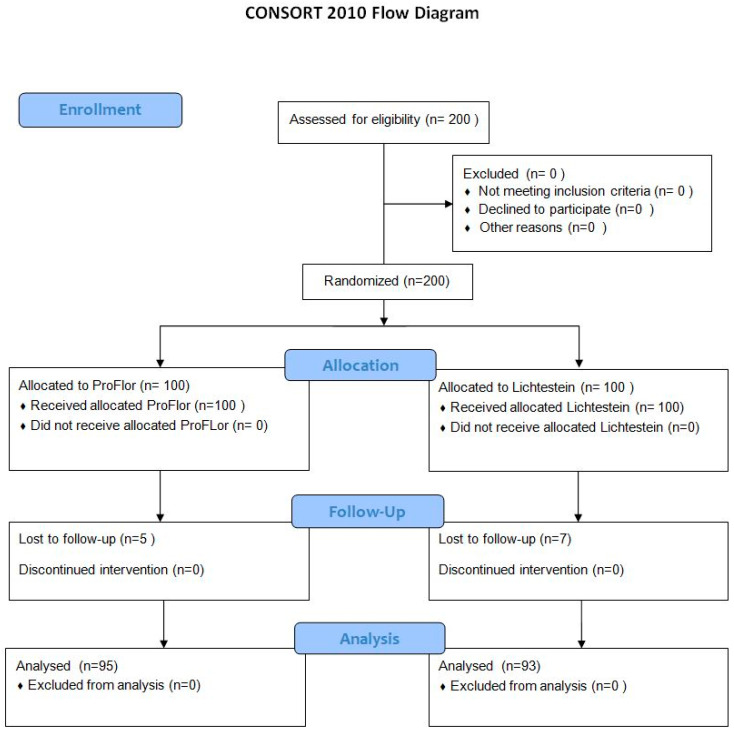
CONSORT 2010 flow diagram.

For this study, institutional ethic approval was granted by University Hospital Policlinico “P. Giaccone” of Palermo. The study has been designed as a multicentric randomized controlled trial and is referred to as the DySLOH (Dynamic Scaffold versus Lichtenstein Open Hernia repair) study registered at ClinicalTrials.gov Identifier: NCT05706662, 31 January 2023. The work has been reported in line with the Consolidated Standards of Reporting Trials (CONSORT) Guidelines (Figure 1) [20].

### 2.1. Patient Selection

Inclusion criteria were individuals aged between 18 and 85 years old, competent to give consent, affected by clinically relevant primary inguinal hernia, scheduled to undergo elective inguinal hernia repair, and eligible for outpatient surgical procedure with local anesthesia. Exclusions criteria are enlisted in Table 1.

### 2.2. Patient Enrollment and Preoperative Schedule

Between February 2021 and May 2022, a total of 200 eligible patients underwent inguinal hernia repair and were included in the study. Before surgery, all patients signed a specific informed consent. The study aimed to compare the outcomes of two surgical techniques for open inguinal hernia repair: the Lichtenstein tension-free technique and the ProFlor technique. The patients were randomly assigned to the two treatment groups, with 100 patients in each arm. A computerized block randomization was performed by our department, and the information regarding planned procedures was sent to the surgical team the day before surgery. The allocated procedure was not concealed to the investigators or patients. During the follow-up period, 12 patients were lost to follow-up, resulting in a final enrollment of 188 patients, divided into the Lichtenstein group (93 patients) and the ProFlor group (95 patients). All of the procedures were performed by teams experienced in hernia surgery using the two different techniques (over 250 procedures). This research received no external funding by institutions or manufacturing firms.

### 2.3. Devices and Surgical Techniques

**Figure 2:**
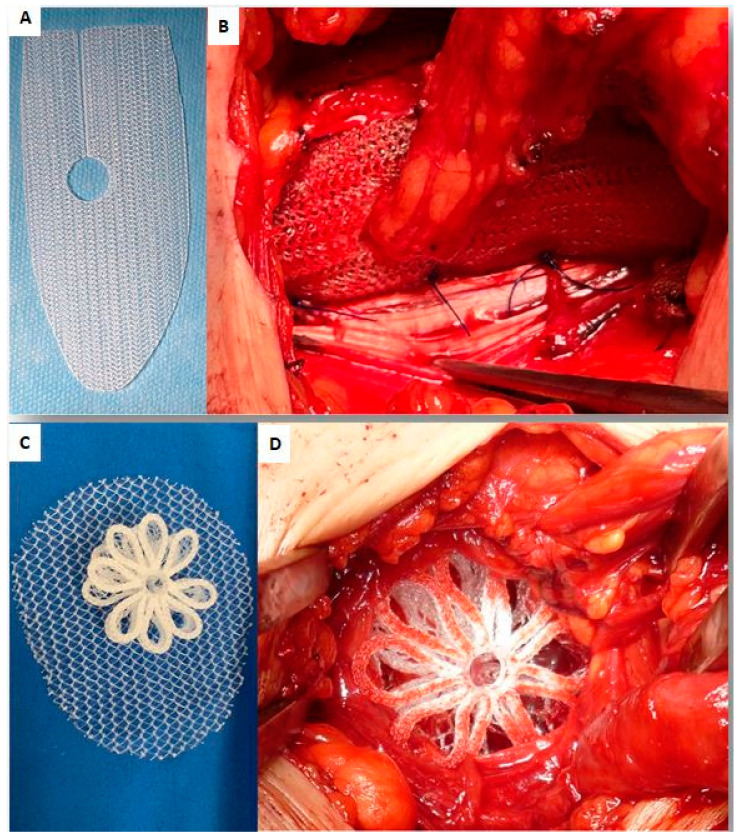
(**A**) A pre-shaped flat mesh for inguinal hernia repair used in the Lichtenstein patient cohort. (**B**) The flat mesh positioned to cover the inguinal floor and fixated with suture stitches to the myotendineal surround. (**C**) The 3D outline of a 40 mm ProFlor with the connected oval flat portion. (**D**) A 40 mm ProFlor scaffold positioned to obliterate an indirect hernia defect.

Both groups of patients underwent the surgical procedure under local anesthesia. The surgeries were performed by two separate groups of surgeons. During the perioperative period, all patients received a single intravenous dose of 2 g of Ceftriaxone. For the Lichtenstein procedures, a lightweight pre-shaped mesh produced by Herniamesh (Herniamesh^®^ S.r.l.|Chivasso (TO)—Chivasso, Italy) was used (Figure 2A).

During the Lichtenstein procedure, the surgical steps involved the following:

1. After making a skin incision, the external oblique fascia was opened. The hernia sac was identified and dissected.
2. In the case of a large indirect hernia, the sac was opened, emptied of its contents, sutured at the base, and cut short. The remaining portion of the sac was then returned to the abdominal cavity. In the case of small sac, this could be returned intact to the abdominal cavity.
3. For direct or combined hernias, the sac was usually not opened and the protrusion was returned back to the abdominal cavity. Then the transversalis fascia was lifted and secured with a continuous polypropylene suture.
4. The mesh was then deployed to cover the anterior aspect of the entire inguinal floor. To accommodate the spermatic cord passing over the mesh, a slit was made in the upper part of the implant. The cord was positioned on the external surface of the mesh and sutured in place.
5. The mesh was fixated without tension on the myotendineal structures of the groin using four to six interrupted stitches made with 2-0 polypropylene monofilament (Figure 2B).
6. Finally, wound closure was performed as usual.

In the ProFlor arm of the study, the inguinal hernias of the patients were repaired using the ProFlor E (Extended) device (Insightra Medical Inc., Clarksville, TN, USA). ProFlor is a 3D scaffold made of low weight, large porous polypropylene material. It features a multilamellar cylindrical core with reinforced edges. The lamellas of the core are 15 mm thick and connected at the center to two polypropylene rings, which give the scaffold a flower-like outline. The 3D core is designed to be compressible on all planes.

The ProFlor E device comes in two sizes: 25 mm, composed of six petals, and 40 mm, which has eight petals. On one surface of the center of the core, ProFlor E is equipped with an oval flat mesh measuring 80 × 100 mm (Figure 2C). This flat portion is intended to be positioned through the hernia defect to counterface the peritoneum. It covers the posterior surface of the inguinal floor, providing a barrier to prevent future protrusions in the remaining parts of the back wall. The ProFlor technique is carried out by a careful exploration of the inguinal floor; after dissecting the hernia sac, the defect is identified and assessed to determine the appropriate size of the 3D scaffold to be used. In the case of an indirect hernia, if the sac is elongated, it is opened and the content returned back to the abdominal cavity; then, the sac is sutured at the base and the redundant portion is cut short. We believe that, in the case of an indirect hernia, creating a small breach in the transversalis fascia to properly position the flat part of the ProFlor device preperitoneally is particularly important in younger patients. This procedural step is designed to prevent the development of a future hernia—whether direct or supravesical—decades later. In the case of direct or supravesical hernias, the transversalis fascia is opened to allow the protrusion to be returned to the abdominal cavity without opening the peritoneal sheath. After the sac is returned, a blunt dissection of the posterior abdominal wall is performed to detach the peritoneal sheath. This dissection creates a wide space to accommodate the flat portion of the ProFlor. Once the inguinal floor is carefully dissected, ensuring the identification of any additional hernia protrusions, a ProFlor device is approached with forceps and positioned at the defect site. The device is then introduced to effectively obliterate the hernial gap (Figure 2D). At this stage, the flat portion of the device is carefully inserted through the hernia defect. It is then deployed and positioned over the posterior aspect of the inguinal floor. After positioning ProFlor, to demonstrate the permanence in situ of the 3D scaffold, a stress test is performed by inviting the patient to cough. This maneuver is performed to confirm that the ProFlor remains securely in place and does not dislodge or shift despite the powerful expulsive force generated during coughing.

During the surgical procedures, detailed intraoperative data were collected, including the type of hernia, size of the defect, and the duration of the operation. Any complications that occurred intraoperatively, such as hematoma, bleeding, vessel injury, as well as any instances of testicular or bowel injury, were carefully documented. In addition to the intraoperative data, qualitative and quantitative information regarding postoperative analgesic treatment was recorded as a discriminating variable between the two patient cohorts. As part of the standard postoperative care, all patients were prescribed oral analgesic treatment with 1000 mg of paracetamol three times a day for the first three days. If patients experienced ongoing pain after this initial period, they were allowed to take an additional 1000 mg of paracetamol as needed. The total consumption of analgesics for each patient was accurately registered, capturing the overall pain management requirements during the postoperative period.

### 2.4. Postoperative Evaluation

All patients included in the study underwent thorough postoperative clinical evaluations and follow-up assessments. These evaluations were conducted at specific time points, including 7 days, 15 days, 1 month, 6 months, 12 months, and 24 months after the surgery. The purpose of these assessments was to identify any potential complications that might have occurred, such as recurrence, hematoma, seroma, testicular swelling, wound complications, infection/abscesses, prosthesis displacement, or any other adverse events.

During each postoperative control visit, patients were also required to complete questionnaires designed to assess their symptoms and overall well-being. For pain assessment in the early postoperative phase (up to one month after surgery), the Visual Analogue Score (VAS) was utilized. However, for long-term assessment beyond one month, patients were asked to complete the Carolinas Comfort Scale (CCS) questionnaire, which was considered the ideal scoring system for evaluating pain, discomfort, and other postoperative symptoms. The CCS questionnaire provided a more comprehensive understanding of the patient’s experience and helped evaluate their long-term outcomes [21,22].

### 2.5. Statistical Analysis

For the statistical analysis, two main primary endpoints were considered between the two cohorts of patients. The first endpoint focused on the overall rate of postoperative complications. The second endpoint of the study focused on assessing postoperative pain and the patient’s quality of life. Two different time frames were considered for this analysis. The first time frame was the early postoperative stage, which extended until one month postoperative. During this period, the intensity of postoperative pain was evaluated using the Visual Analogue Scale (VAS) score. The second time frame was the late postoperative phase, which spanned from one month to two years postoperative. During this period, the assessment of postoperative pain and the patient’s quality of life was conducted using the Carolinas Comfort Scale (CCS). The statistical analysis was conducted using SPSS 25.0 (SPSS Inc., Chicago, IL, USA). Quantitative variables were presented as mean values with standard deviation and were compared using the Student’s *t*-test. Categorical variables were presented as counts and percentages and were compared using the Chi-squared test. A *p*-value < 0.05 was considered statistically significant. Before enrolling patients in the two groups, an adequate power analysis was performed to ensure that the study had sufficient statistical power and to avoid Type II errors. The power analysis determined that a minimum of 83 patients would be required to detect a clinically significant difference, such as a reduction of 2 points in the VAS score for postoperative outcomes between the two groups [5,6,7,8,9,10,11,12]. The significance level was set at 5%, and the power calculation was set at 80%. By conducting the power analysis, the study aimed to ensure that the sample size was appropriate to detect significant differences between the ProFlor and Lichtenstein groups, providing reliable and meaningful results.

## 3. Results

**Figure 3:**
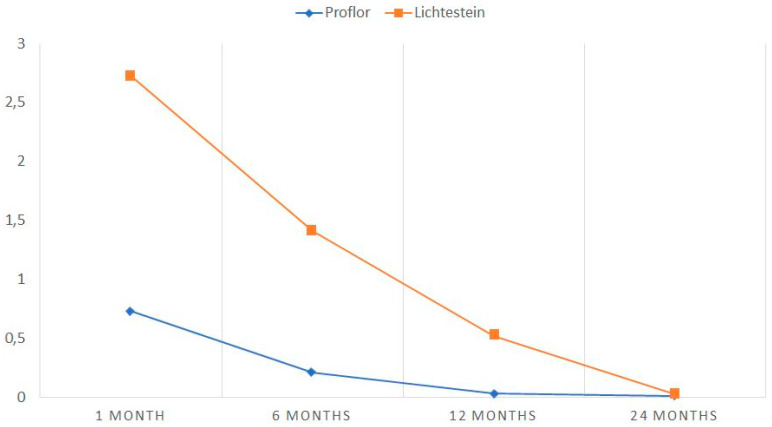
CCS scores at 1, 6, 12, and 24 months, comparing Lichtenstein and ProFlor techniques. On the Y-axis, we report the mean values of the CCS scores as listed in Table 6.

All patients included in the study, whether in the Lichtenstein or ProFlor groups, underwent surgery as outpatients in a clinical setting during the specified period. Regarding patient demographics, there were nine female patients for one hundred seventy-nine male patients, resulting in a female-to-male ratio of 9:179. The mean age of the patients was 59.7 years ± 33. Out of the total cohort, 85 patients (45.2%) had other comorbidities (primarily hypertension, diabetes mellitus, or dyslipidemia). Further information on patient demographics and clinical data can be found in Table 2. Regarding the classification of hernias observed during surgery in the examined cohorts, different classifications were utilized based on the surgical technique employed. In the Lichtenstein group, we utilized the EHS classification and identified 46 patients (49.4%) with indirect inguinal hernias, 34 patients (36.5%) presented with direct hernias, and 13 patients (14%) had combined hernias. For the ProFlor group, the technique focuses on selectively obliterating the hernia opening, necessitating a thorough examination of the inguinal floor to avoid overlooking any ipsilateral hernias. Since current classification lists do not include all protrusion types found, like multiple ipsilateral and supravesical hernias, the protrusions were categorized based on a novel updated classification that also includes these types [23]. In detail, we observed the following hernia types in the ProFlor group: thirty-nine patients (41%) had indirect hernias, eighteen patients (18.9%) had direct hernias, twelve patients (12.6%) had multiple ipsilateral hernias, nineteen patients (20%) had combined hernias, and seven patients (7.3%) had supravesical protrusions. Surgical procedures for the ProFlor group had a mean duration of 42 min (SD ± 15.4), while the Lichtenstein group had a mean operative time of 56 min (SD 24.6, *p*-value < 0.028). Further details regarding hernia type defect size and the duration of each surgical procedure can be found in Table 3. Regarding intraoperative complications, only one patient who underwent Lichtenstein hernia repair experienced an episode of intraoperative bleeding from the epigastric vessels. However, this bleeding was promptly controlled. No intraoperative complications were observed during ProFlor procedures. In terms of follow-up, seven patients (7.5%) in the Lichtenstein group and five patients (5.3%) in the ProFlor group did not respond to the 6-month follow-up and subsequent scheduled appointments. Regarding early postoperative complications within one month after surgery, seroma was the most frequent occurrence, with six cases (6.4%) in the Lichtenstein group and three patients (3.1%) in the ProFlor group. Four cases of hematoma (4.3%) occurred in the Lichtenstein group, while no hematomas were reported in the ProFlor group. Additionally, one patient who underwent Lichtenstein hernia repair experienced a wound infection. In contrast, no wound infection was reported in patients of the ProFlor group. No cases of recurrence occurred during the early stage in both cohorts. Overall, the occurrence of early postoperative complications (including seroma, hematoma, and infections) was eleven cases (11.8%) in the Lichtenstein group and three cases (3.1%) in the ProFlor group, indicating a statistically significant difference (*p*-value = 0.029). No cases of implant dislodgement, recurrences, or other adverse events were observed during the early postoperative period. Regarding complications in the late postoperative stage (beyond one month), eleven patients (11.8%) who underwent Lichtenstein hernia repair reported chronic pain. Among these cases, three resolved within six months with medical therapy, but the remaining cases continued to experience pain even beyond the duration of the investigation. Three patients in the Lichtenstein group experienced testicular swelling, which was successfully managed with conservative therapy and resolved within six months. Four patients of the Lichtenstein cohort experienced recurrence within two years postoperative. Conversely, apart from the previously mentioned cases of seroma, no significant late complications and no recurrence were observed in patients treated with the ProFlor technique. Further information on postoperative complications for both examined techniques can be found in Table 4. The study results indicate that patients who underwent the ProFlor technique experienced significantly lower levels of early postoperative pain compared to those treated with the Lichtenstein technique. On the day of discharge, ProFlor patients had an average pain score of 2.6 on the Visual Analogue Scale (VAS), whereas Lichtenstein patients had a higher average score of 5.7, indicating more intense pain. By ten days postoperative, the majority of ProFlor patients were completely pain-free, with a mean VAS score of 0.2. In contrast, Lichtenstein patients still reported some pain, with a mean VAS score of 3.1. These differences in pain scores persisted even at 30 days postoperative, at which point all ProFlor patients reported being painless, while Lichtenstein patients had an average VAS score of 1.8 (Table 5). Notably, a subset of Lichtenstein patients (16 out of 93) continued to experience pain scores equal to or greater than two, indicating persistent pain or recurrent discomfort. In terms of long-term pain assessed using the CCS scoring scale, patients who underwent the Lichtenstein technique had an overall score of 61.17 (mean 2.66) after one month. Conversely, ProFlor patients reported a significantly lower overall score of 16.90 (mean 0.73), indicating a considerable disparity in terms of sensation, movement limitation, and pain between the two employed techniques. The comparative overall CCS scores for Lichtenstein and ProFlor patients at subsequent stages were as follows: 33.08 (mean 1.44) for Lichtenstein and 5.04 for ProFlor (mean 0.21) at 6 months; 11.35 for Lichtenstein (mean 0.49) and 0.75 for ProFlor (mean 0.003) at 12 months; and 0.55 for Lichtenstein (mean 0.02) and 0.18 for ProFlor (mean 0.008) at 24 months. In the postoperative period, one month after surgery, the likelihood of experiencing symptoms in the pain and limitations subscale of CCS was found to be higher in univariate analysis when the traditional Lichtenstein repair (with sutured mesh) was used (odds ratio [OR] 5.265, 95% confidence interval [CI] 1.754–12.498; *p* < 0.001). Furthermore, multivariate analysis supported this finding specifically for patients who underwent the classical Lichtenstein repair with sutured mesh. Table 6 reports the average scores and the trend over time of the Carolinas Comfort Scale for the two cohorts of patients undergoing inguinal hernioplasty. Since a CCS score below two is considered as not clinically significant, it becomes evident that with the use of the ProFlor scaffold, this threshold is already achieved within 1 month from the surgery. On the other hand, patients undergoing the Lichtenstein technique have to wait for 6 months postoperatively to reach this result. Table 6 and Figure 3 summarize the results of the CCS scoring system.

## 4. Discussion

**Figure 4:**
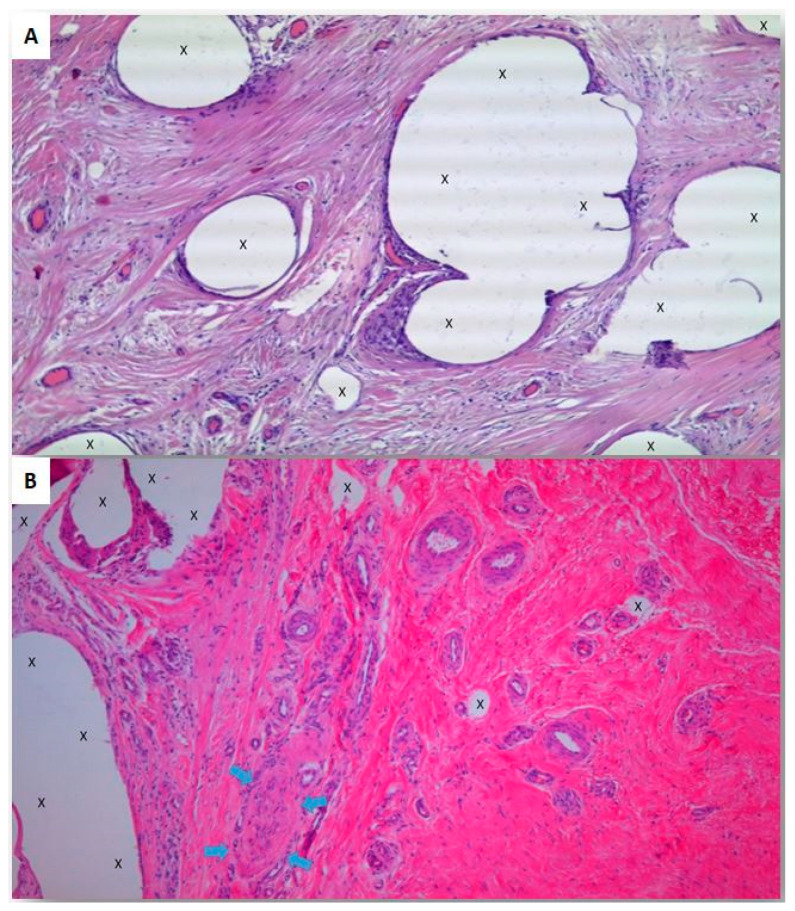
(**A**) Biopsy specimen from flat mesh explanted for the recurrence of inguinal hernia 8 months postoperative: In the areas immediately adjacent to the implant fibers of the flat mesh (X), a productive chronic inflammatory infiltrate is observed, including some foreign body giant cells. Rare vascular structures, primarily consisting of small capillaries, are present in the adjacent areas as an accompanying phenomenon to chronic inflammation. Overall, the surface of the section is mainly characterized by the unregulated formation of fibrotic scar tissue (E&E 200X). (**B**) Biopsy specimen excised from ProFlor 7 months post-implantation during Winkelmann procedure for hydrocele: Multiple blood vessels are observed, represented by well-formed arteries and veins, in the late phase of growth adjacent to the implant fibers polypropylene material of the 3D framework (X). There is an absence of noticeable inflammatory response. A muscle bundle in the stage of progressive evolution is observed close to the implant fibers, dispersed among stratified and well constituted connective tissue. Close to the implant fibers is present a nervous structure showing axons and related myelin sheath in a late stage of development (light blue arrows) (E&E 200X).

The Lichtenstein open anterior approach, the most popular inguinal hernia repair technique, involves the deployment of flat meshes over the herniated groin with the aim of reinforcing the inguinal floor. However, this surgical approach raises some controversial issues, including mesh fixation, defect patency, and poor quality biological response. The literature has linked mesh fixation to various postoperative complications, such as tissue tears, bleeding, hematomas, mesh displacement, discomfort, and chronic pain [23,24,25]. Chronic pain, in particular, is a concerning complication that significantly affects the patient’s quality of life and is considered a specific adverse event associated with the Lichtenstein technique [26,27]. To avoid the need for point fixation, fibrin glue has been explored as an alternative method for securing flat meshes [28,29,30,31]. Due to its short period of efficacy, the use of fibrin glue does not appear to be an effective method for mesh fixation [32]. Another aspect of Lichtenstein repair that is often overlooked, pertains to the management of the hernial defect. Following mesh placement, the hernial opening remains open. To address the patency of the hernial opening, a variation of the Lichtenstein technique, known as plug and mesh repair, involves inserting a plug into the defect. To prevent migration, it is considered essential to secure the plug to the deep ring [33,34]. Another significant concern in Lichtenstein hernia repair is related to the biological response of flat meshes. Typically, within a few months, a rigid fibrotic scar plaque forms and incorporates the static and passive meshes, resulting in a surface reduction of at least 20–30% due to shrinkage [5]. The combination of the rigid mesh and scar tissue limits groin movements and causes friction against the inguinal structures, leading to long-term discomfort (chronic pain syndrome) [35]. In addition to the well-known issues, a recent line of thought has examined current concepts in inguinal hernia repair from a pathogenetic perspective. Several scientific investigations have demonstrated that inguinal protrusion disease is caused by progressive tissue degeneration resulting from chronic compressive injury [27]. This implies that a pathogenetically coherent approach to treatment should aim to stop degeneration and promote the regeneration of the weakened inguinal structures. Considering the treatment concept of reinforcing the groin with flat meshes, as seen in the Lichtenstein technique, it is noteworthy that the foreign body reaction triggered by the mesh leads to fibrotic scarring (Figure 4A). However, this response resembles a regressive rather than a regenerative biological process. In summary, the biological response to flat meshes in hernia repair falls short of healing the degenerative nature of the disease and fails to achieve the goal of restoring the integrity of the inguinal barrier by regenerating the essential tissue components of the groin. To address the inherent incongruences of the Lichtenstein technique and its variants, extensive research focused on the physiological and pathogenetical aspects has led to the development of the surgical treatment of inguinal protrusions using a 3D dynamic compliant scaffold called ProFlor, which when experimentally tested demonstrated promising results [27]. Due to its dynamic nature, the 3D scaffold moves synchronously with the groin and remains imperceptible to patients. Importantly, ProFlor is compliant with the cyclical load of the groin, triggering a probiotic biological response through specific tissue growth factors attracted by the movements of the scaffold [17,36,37]. This response promotes the development of newly formed muscles, vessels, and nerves, essential tissue components of the groin that can also be seen with an MRI [38,39]. Within a few weeks, a viable fleshy structure incorporates the 3D scaffold, fully restoring the integrity of the herniated inguinal barrier (Figure 4B). Given the homogeneous patient population in both groups and the nearly equivalent recurrence rate, the analysis of the collected raw data and the corresponding statistical analysis highlight the superiority of the ProFlor technique. Notably, the Lichtenstein approach exhibits significantly longer procedure times and a higher incidence of intraoperative complications compared to ProFlor. In this regard, we would like to point out that in both the Lichtenstein method and other pure tissue repair techniques, bleeding often results from tissue tearing associated with suture application. Our report tries to emphasize that a fixation-free technique like ProFlor leads to fewer instances of bleeding compared to methods where the myotendineal structure of the groin is manipulated with sutures. The straightforward and efficient placement of the 3D scaffold without the need for fixation explains these favorable outcomes. On the other hand, the requirement for mesh fixation in the Lichtenstein procedure extends the mean duration of the surgery. It is also Important to emphasize that the process of mesh fixation may lead to intraoperative complications such as tissue tearing and subsequent bleeding during suture knot tying. The occurrence of such mishaps during mesh fixation can result in significant time wastage and an increased postoperative consumption of painkiller. Another undervalued aspect concerns the precise acknowledgement of the encountered hernia types, but this specific aspect seems not to be taken into consideration during Lichtenstein hernia repair. For the purpose of categorizing the hernia types encountered in ProFlor patients, a recent classification list that includes these hernia types and organizes the protrusions based on the inguinal fossae from which they originate was utilized [23]. At this point, it is worth noting that the use of the ProFlor scaffold requires the precise dissection of the inguinal floor to identify multiple protrusions within the same groin. Indeed, the ProFlor technique is designed to address the selective obliteration of the hernia defect. In cases where multiple protrusions exist within the same groin, it is essential to manage each additional hernia individually, often requiring the placement of an additional ProFlor scaffold in the second defect. Continuing the comparative evaluation, it becomes apparent that the Lichtenstein approach exhibits a higher rate of early postoperative complications, approximately 27% more, particularly those related to mesh fixation, such as bleeding, hematoma, discomfort, and chronic pain. In contrast, these types of complications did not occur in the ProFlor cohort due to the fixation-free procedure, which results in a shorter operative time and reduced trauma. Among the patients in the ProFlor group, the only type of early complication observed was the development of seroma. When comparing the two techniques, the rate of recurrence within one year strongly favored the ProFlor approach, with no reported recurrences, in contrast to the four observed in the Lichtenstein group. This notable difference can be attributed to a specific factor. Following a thorough inspection of the inguinal floor to exclude any additional ipsilateral protrusions, the ProFlor technique achieves a permanent obliteration of the hernia defect, which can be intraoperatively confirmed by conducting a stress test by coughing after the scaffold is deployed into the hernia opening. On the other hand, recurrence within 24 months after Lichtenstein repair can occur due to various factors. One significant factor is the possibility of tissue tear caused by suture stitches, which can result in the mesh becoming unfastened and a re-protrusion through the original defect. Additionally, the presence of forgotten ipsilateral protrusions can contribute to the development of recurrence in the early/mid postoperative stages. Regarding the comparative evaluation of early postoperative pain using the Visual Analogue Scale, significant differences were observed between the two techniques. The collected data clearly indicated a higher prevalence of significant pain in patients who underwent Lichtenstein repair. In contrast, a significant majority of patients who underwent the ProFlor technique reported being pain-free starting from one week postoperative, and an even higher percentage experienced pain relief within 10 days. These results demonstrate a substantial improvement in postoperative pain outcomes for patients treated with the ProFlor technique compared to those undergoing the Lichtenstein repair. Regarding the return to daily activities, a notable disparity emerges between the two techniques. Within one week, 94% of ProFlor patients were able to fully resume their normal way of life, whereas none of the Lichtenstein patients were able to do so in the same time frame. Moving on to the assessment of long-term symptoms in the late postoperative stage, the evaluation of the 23 points of the CCS likely confirmed the superiority of the ProFlor technique. Specifically, only the Lichtenstein group reported individuals experiencing long-term pain and discomfort or reported consistent movement-induced pain that negatively impacted their quality of life. On the other hand, no discomfort was reported by patients who underwent the ProFlor technique, further emphasizing its advantages in terms of postoperative comfort and mobility. The high rate of discomfort experienced by patients treated with the Lichtenstein technique can be attributed to the development of a granuloma, which is a result of poor-quality tissue incorporation and the stiff scar plate formed by the implanted flat mesh. In contrast, the fixation-free deployment and probiotic biological response of ProFlor eliminate the occurrence of such complications from the beginning. Specifically, these events do not occur in ProFlor patients because the 3D hernia scaffold moves in harmony with the groin, adapting to the natural cyclical load of the inguinal area and ensuring compliance with the dynamic nature of the region. The motile behavior of ProFlor activates a probiotic biological response that, unlike flat meshes, leads to the development of new muscular, vascular, and nervous elements in the 3D scaffold. This unique regenerative biologic response seems to be the reason why no chronic pain and no discomfort have been reported by patients operated on with ProFlor. However, the study does have two significant limitations. The first limitation is the follow-up period, which should ideally be extended to at least five years. We are planning to address this limitation in a future study. Second, all patients were treated by surgeons experienced in hernia treatment. In clinical practice, we have to consider the physiological learning curve of a new surgical technique. Another possible limitation of this study regards the cost of the procedure. The company’s pricing policy varies by country but we estimate that the ProFlor scaffold, for its specific characteristics, is more expensive than the classical flat mesh used in the Lichtenstein repair. At the same time, we think this information is not directly related to this scientific research that evaluates not the economic cost but the biological response and the clinical outcomes of the surgical procedure.

## 5. Conclusions

The results of this investigation appear to demonstrate that the ProFlor technique likely surpasses the conventional Lichtenstein approach in terms of intraoperative, short-term, and long-term outcomes. Considering the seeming superiority of ProFlor and the limitations associated with the Lichtenstein technique, it is important for the surgical community to reflect on the conventional approach based on reinforcing the herniated groin with static and fixated meshes. With the advancements in our understanding of the pathophysiology and development of inguinal protrusion disease, it may be necessary to reevaluate whether the current techniques aligns with the scientific progress achieved over the recent years.

## Figures and Tables

**Table 1:** Exclusion criteria.

Exclusion Criteria
Recurrent inguinal hernia Incarcerated inguinal hernia Hernia not in the inguinal area Signs of obvious local or systemic infection ASA score > 4 Presenting with unstable angina or NYHA class of IV Pregnant Active drug user Immunosuppression, chemotherapy Chronic renal insufficiency Abdominal ascites Infection in the area of the surgical field BMI > 35

**Table 2:** Patient demographics, comorbidities, and ASA scores.

	*Lichtenstein (n = 93)* *Mean* ± *SD*	*ProFlor (n = 95)* *Mean* ± *SD*	*Total (n = 188)* *Mean ± SD*	*p-Value*
***Age***	*Years* 59.7 ± 32	*Years* 59.6 ± 33.2	*Years* 59.7 ± 33	*1.000*
***Gender***				
*M*	86 (92.5%)	93 (97.9%)	179 (95.2%)	*0.802*
*F*	7 (7.5%)	2 (2.1%)	9 (4.7%)	*0.272*
***Body Mass Index***	26.8 ± 5.6	27 ± 7.8	26.9 ± 7.8	*0.998*
***Comorbidity***	45 (48.4%)	40 (42.1%)	85 (45.2%)	0.719
*Cardiovascular*	26 (28%)	28 (29.4%)	54 (28.7%)	
*Pulmonary*	6 (6.4%)	2 (2.1%)	8 (4.2%)	
*Diabetes*	6 (6.4%)	5 (5.3%)	11 (5.8%)	
*Others*	7 (7.5%)	5 (5.3%)	12 (6.4%)	
***ASA score***				
*ASA 1*	48 (51.6%)	55 (57.8%)	103 (54.8%)	*0.667*
*ASA 2*	36 (38.7%)	34 (35.8%)	70 (37.2%)	*0.576*
*ASA 3*	9 (9.7%)	6 (6.3%)	15 (8%)	*0.562*

**Table 3:** Hernia types, defect size (overall/percentage), and duration of the surgical procedures.

	Lichtenstein (*n* = 93)	ProFlor (*n* = 95)	Total (*n* = 188)	*p*-Value
**Hernia types**	***Overall (%)***	***Overall (%)***	***Overall (%)***	
Indirect	46 (49.4%)	39 (41%)	88 (46.8%)	0.37
Direct	34 (36.5%)	18 (18.9%)	53 (28.2)	**0.021**
Combined	13 (14%)	19 (20%)	32 (17%)	0.59
Supravesical	NA	7 (7.3%)	7 (3.7%)	NA
Multiple ipsilateral	NA	12 (12.6%)	12 (6.7%)	NA
**Side**				
Right	51 (54.8%)	51 (53.7%)	102 (54.2%)	0.818
Left	42 (45.2%)	44 (46.3%)	86 (45.7%)	0.818
**Defect size (cm)**	2.4 ± 1.5	2.8 ± 1.8	2.6 ± 1.85	0.684
***Procedure duration***	56 min ± 24.6	42 min ± 15.4	48.5 min ± 19.2	**0.028**

**Table 4:** Postoperative complications comparing Lichtenstein/ProFlor.

	*Lichtenstein (n = 93)* *n. (%)*	*ProFlor (n = 95)* *n. (%)*	*p-Value*
***Intraoperative complications***	1 (1.1%) (epigastric vessel bleeding)	-	0.998
***Early complications***			
Seroma	6 (6.4%)	3 (3.1%)	
Hematoma	4 (4.3%)	-	
Wound infection	1 (1.1%)	-	
Recurrence	0 (0%)	0 (0%)	
Early complications (all)	11 (11.8%)	3 (3.1)	**0.029**
***Late complications***			
Chronic pain	11 (11.8%)	0 (0%)	
Testicular swelling	3 (3.2%)	0 (0%)	
Recurrence	4 (4.3%)	0 (0%)	
Late complications (all)	18 (19.3%)	0 (0%)	**0.002**

**Table 5:** VAS scores at postoperative day 0, 10, and 30.

VAS Scores POD 0/10/30			
	***Lichtenstein (n = 93)*** ***Mean* ± *SD***	***ProFlor (n = 95)*** ***Mean* ± *SD***	***p-Value***
POD O	5.7 ± 1.9	2.6 ± 0.8	**0.016**
POD 10	3.1 ± 1.7	0.2 ± 0.6	**0.016**
POD 30	1.8 ± 1.2	-	0.634

**Table 6:** Carolinas Comfort Scale scores comparing mean values obtained for 23 items between Lichtenstein and ProFlor at 1, 6, 12, and 24 months.

	1 Month *Lichtenstein/ProFlor*	6 Months *Lichtenstein/ProFlor*	12 Months *Lichtenstein/ProFlor*	24 Months *Lichtenstein/ProFlor*
***Laying down***				
A. sensation of mesh	2.61/1.15	1.26/0.18	0.28/0.01	0.01/0.00
B. pain	2.12/0.91	1.15/0.20	0.15/0.00	0.00/0.00
***Bending over***				
A. sensation of mesh	3.10/1.0	1.30/0.25	0.54/0.00	0.03/0.01
B. pain	2.42/0.81	1.56/0.22	0.41/0.01	0.02/0.00
C. movement limitations	2.63/0.82	1.85/0.36	0.52/0.10	0.02/0.00
***Sitting***				
A. sensation of mesh	3.05/0.77	1.23/0.32	0.50/0.01	0.02/0.01
B. pain	2.09/0.75	1.12/0.31	0.42/0.06	0.01/0.01
C. movement limitations	2.61/0.82	1.85/0.28	0.51/0.05	0.04/0.03
***Activities of daily living***				
A. sensation of mesh	2.45/0.75	1.05/0.15	0.44/0.02	0.05/0.01
B. pain	2.95/0.67	1.52/0.23	0.36/0.03	0.03/0.01
C. movement limitations	2.84/0.71	1.95/0.20	0.36/0.10	0.04/0.00
***Coughing or deep breathing***				
A. sensation of mesh	2.25/0.68	1.25/0.32	0.69/0.06	0.03/0.02
B. pain	3.12/0.83	1.38/0.24	0.70/0.03	0.02/0.02
C. movement limitations	2.75/0.64	1.44/0.20	0.70/0.04	0.04/0.01
***Walking***				
A. sensation of mesh	3.15/0.55	1.18/0.16	0.32/0.02	0.01/0.00
B. pain	2.78/0.50	1.20/0.14	0.43/0.03	0.01/0.01
C. movement limitations	2.67/0.51	1.29/0.14	0.48/0.03	0.00/0.00
***Stairs***				
A. sensation of mesh	2.08/0.66	1.62/0.21	0.56/0.030	0.02/0.02
B. pain	2.80/0.52	1.35/0.17	0.50/0.02	0.02/0.01
C. movement limitations	2.72/0.54	1.32/0.18	0.51/0.00	0.01/0.00
***Exercise***				
A. sensation of mesh	2.10/0.51	1.80/0.14	0.87/0.01	0.06/0.00
B. pain	2.63/0.85	1.42/0.21	0.55/0.01	0.04/0.01
C. movement limitations	3.25/0.95	1.99/0.23	0.55/0.08	0.02/0.00
**Overall**	61.17/16.90	33.08/5.04	11.35/0.75	0.55/0.18
**Mean**	2.66/0.73	1.44/0.21	0.49/0.03	0.02/0.008

## Data Availability

The datasets used and/or analyzed during the current study are available from the corresponding author on reasonable request. All data generated or analyzed during this study are included in this published article.
